# Clinical Evaluation of a Centralized In-Clinic Direct Culture and Antimicrobial Susceptibility Testing Workflow for Canine and Feline Skin, Ear, and Urine Infections

**DOI:** 10.3390/antibiotics15101016

**Published:** 2026-10-13

**Authors:** Blaž Cugmas, Eva Štruc, Urška Vesel, Daira Viškere, Liisi Kulasalu, Dulmini Nanayakkara Sapugahawatte

**Affiliations:** 1 Vetamplify SIA, LV-1011 Riga, Latvia; 2 Faculty of Science and Technology, University of Latvia, LV-1004 Riga, Latvia; 3 Prva-K, Small Animal Clinic, SI-1000 Ljubljana, Slovenia; 4 Faculty of Veterinary Medicine, Latvia University of Life Sciences and Technologies, LV-3004 Jelgava, Latvia; 5 Institute of Veterinary Medicine and Animal Science, Estonian University of Life Sciences, 51014 Tartu, Estoniadulmini.sapugahawatte@emu.ee (D.N.S.)

**Keywords:** direct culture, antimicrobial susceptibility testing, chromogenic Mueller–Hinton agar, disc diffusion, point-of-care testing, pyoderma, otitis externa, urinary tract infection, dogs and cats

## Abstract

Background/Objectives: Rapid antimicrobial decision-making in small-animal practice is limited by the turnaround time (≥2 days) of conventional laboratory-based culture and antimicrobial susceptibility testing (AST). Existing point-of-care and in-clinic tests can provide faster results, but their performance often depends on user experience. This study evaluated VetBac, a hybrid workflow in which the test is conducted in the clinic, while experts centrally interpret bacterial growth on plates. Methods: We analyzed 60 interpretable plates from canine and feline skin, ear, and urine infections. In the clinic, samples were inoculated onto chromogenic Mueller–Hinton agar plates, after which antibiotic discs were applied. Plates were incubated for 16–20 h, photographed, and interpreted centrally by an expert. For comparison, samples were submitted in parallel to standard microbiology laboratories. Results: Identification of primary bacterial populations in clinical samples showed high species-level agreement with laboratory results (83.9%), increasing to 100.0% when staphylococci species were grouped. Secondary bacteria agreement (16.7%) was lower due to unrecognizability and overgrowth by dominant bacteria. Overall AST agreement was 93.6% (262/280), with 91.9% sensitivity for detecting resistance and 94.0% specificity for detecting susceptibility. VetBac-susceptible discrepancies occurred in 5/62 laboratory-resistant comparisons (8.1%), and VetBac-resistant discrepancies occurred in 13/218 laboratory-susceptible comparisons (6.0%). Conclusions: In interpretable skin, ear, and urine samples, the VetBac workflow showed high agreement with laboratory results for primary bacterial identification and AST. These findings support the hybrid in-clinic/centralized model as an adjunctive source of earlier antimicrobial decision support, pending laboratory confirmation. However, the study did not estimate the proportion of all clinical samples suitable for direct culture and AST.

## 1. Introduction

Small-animal urinary tract (UTIs), skin (pyoderma), and ear infections (otitis) are common clinical situations in which antimicrobial treatment is considered, and together they account for a substantial proportion of antimicrobial prescriptions in dogs and cats [1,2,3]. Current guidelines on UTIs and pyoderma [4,5] thus increasingly emphasize a diagnostic and stewardship-based approach in which antimicrobial treatment is supported by evidence of bacterial infection and, when systemic therapy is required, by bacterial culture and antimicrobial susceptibility testing (AST).

For suspected bacterial cystitis, aerobic bacterial urine culture is preferred because urinalysis and sediment examination do not provide definitive evidence of infection [4]. This is particularly relevant in cats, as bacterial UTIs account for a minority (~15%) of lower urinary tract disease cases [6,7]. In contrast, positive cultures were present in around 45% of suspected canine cases [7,8]. Therefore, empirical antimicrobial treatment can be justified in dogs with suspected sporadic cystitis, when previous antimicrobial exposure is limited, and likely pathogens and susceptibility patterns are predictable [4]. For pyoderma, the guideline [5] recommends cytology to confirm bacterial involvement and emphasizes topical antimicrobial therapy, including antiseptics, as the preferred approach for many surface and superficial infections. Systemic antimicrobials are reserved for selected infections, such as deep pyoderma, and also superficial infections when topical therapy is not feasible or insufficient. In these cases, culture and AST are recommended to guide antimicrobial selection [5].

Conventional laboratory culture and AST remain the reference diagnostic approach for confirming bacterial infection and guiding antimicrobial treatment. However, because external laboratory turnaround times are long (≥2 days) and costs can be high, empirical treatment can outweigh routine use of culture and AST in veterinary practices. For this reason, several in-clinic phenotypic approaches, including culture-based point-of-care (PoC) tests, have been developed to support faster bacterial detection, identification, or antimicrobial susceptibility assessment in small-animal practice (Table 1). Other rapid approaches, such as bacteriuria immunoassays [9,10] or targeted molecular tests for methicillin-resistant *Staphylococcus pseudintermedius* (MRSP) [11], have also been investigated, but these generally provide specific rather than broad bacterial identification and phenotypic AST.

Published studies [12,13,14,15,16,17] generally report good performance in detecting bacteriuria, with sensitivities and specificities above 80%. A similar trend was observed for AST, with reported sensitivity, specificity, and agreement with laboratories ranging from about 80 to 95%. In contrast, bacterial identification (ID) is more variable and appears strongly dependent on evaluator experience. The reported ID agreements ranged from about 50% in less experienced users to near-perfect agreement when experts interpreted results [13,14,15].

As the PoC tests performance often depends on user experience, the present study evaluated the VetBac platform (VetBac SIA, Riga, Latvia), a hybrid in-clinic/centralized interpretation system. In VetBac workflow, samples are inoculated and incubated in the clinic, while plate images are uploaded and interpreted centrally by veterinary microbiology-trained reviewers. VetBac-based bacterial identification and AST results were compared with routine diagnostic laboratory results from canine and feline samples collected from urinary tract, skin, and ear infections. This work was an exploratory agreement study of interpretable samples, rather than a diagnostic yield study of all clinical samples submitted with suspected infection.

## 2. Results

The available final agreement dataset included 60 interpretable VetBac plates (Table 2) from skin (*n* = 25), ear (*n* = 11), and urinary tract samples (*n* = 24). Most samples came from dogs (*n* = 53), with fewer from cats (*n* = 7). According to the laboratory comparator approach, 32 samples were classified as clinical sample comparisons and 28 as plate isolate comparisons. These samples represented cases in which sufficient growth and plate quality allowed bacterial identification and AST interpretation; the study was not designed to estimate the proportion of all submitted clinical samples that would meet these criteria.

### 2.1. Culture

**Figure 1:**
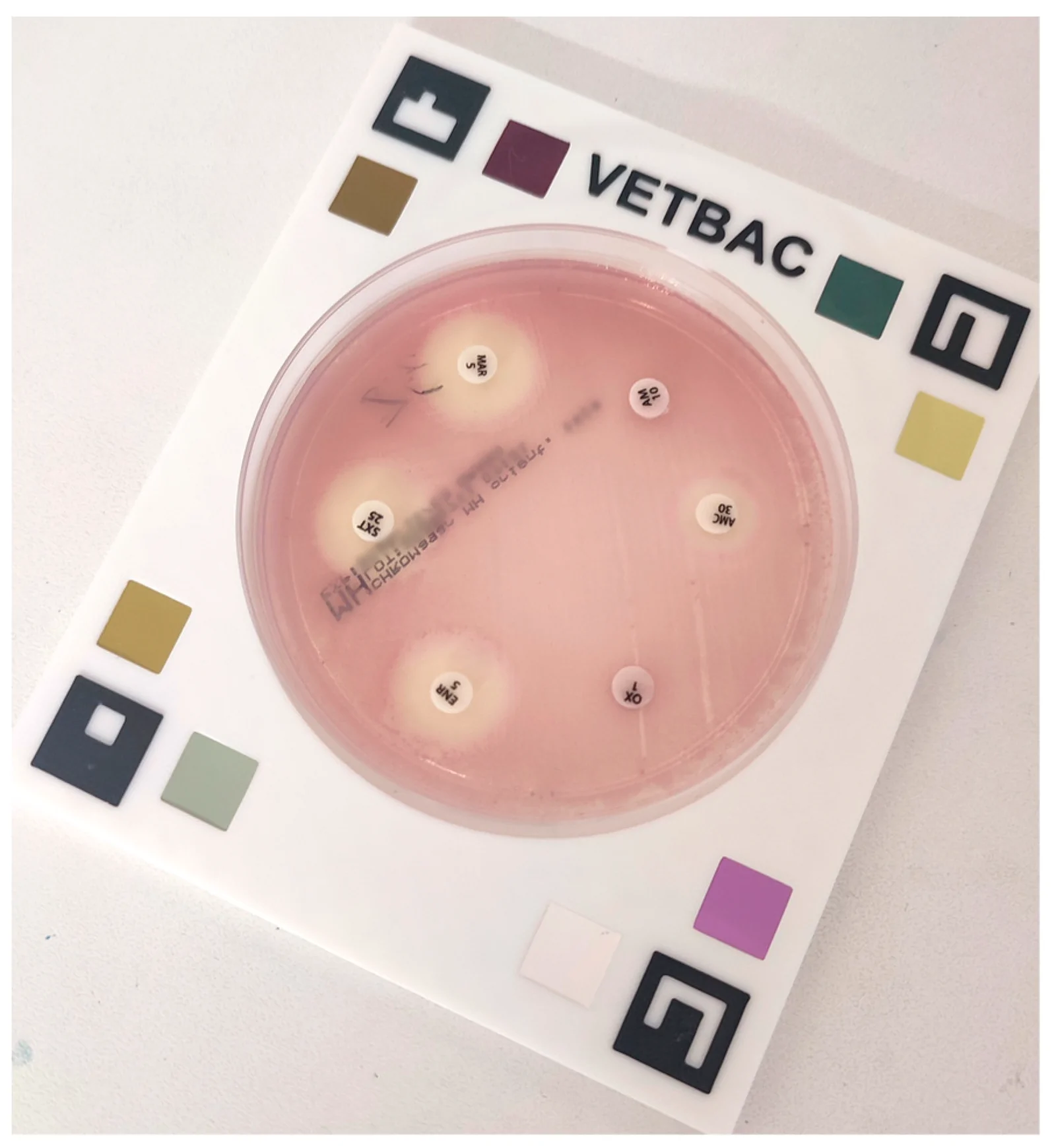
Representative VetBac plate showing *Escherichia coli* growth on chromogenic Mueller–Hinton agar after direct inoculation of a clinical sample and overnight incubation.

Laboratory bacterial identification showed a distribution consistent with the sampled infection types. The dataset was dominated by common canine and feline skin, ear, and urinary tract bacteria (Table 3), especially staphylococci and Enterobacterales (Figure 1).

**Figure 2:**
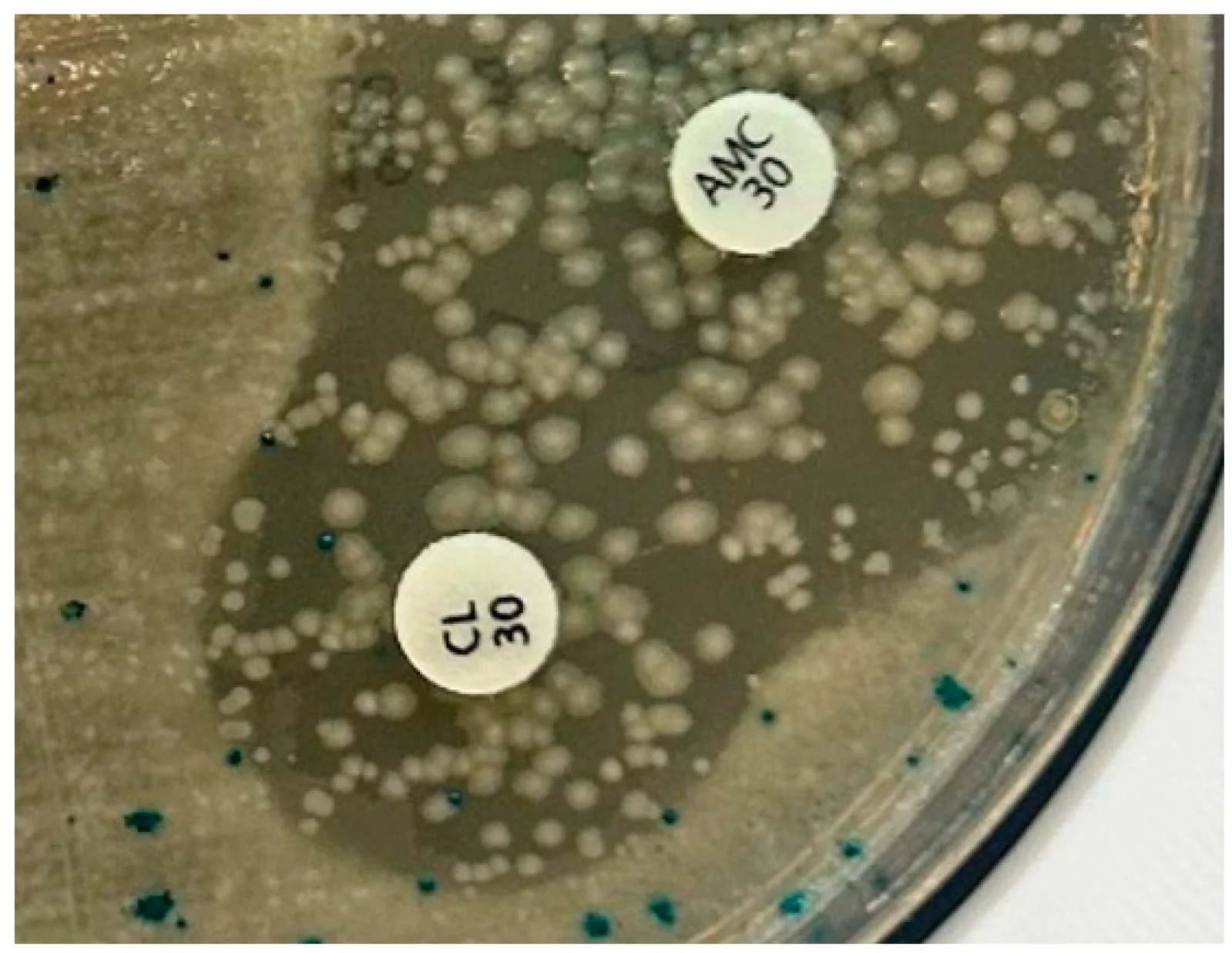
Direct ear culture on chromogenic Mueller–Hinton plate showing mixed growth of *Pseudomonas aeruginosa* (separate large colonies around the AMC [amoxicillin–clavulanate] and CL [cephalexin] discs) and *Proteus mirabilis* (diffuse spreading growth).

In two clinical samples, the laboratory did not report the same primary clinically relevant bacterial finding. In one urine sample with plate-isolated *S. pseudintermedius*, the laboratory reported no bacterial growth. Thus, we excluded the sample from further analysis as no comparable laboratory organism or AST result was available. In one ear sample, we detected *Pseudomonas aeruginosa* together with *Proteus mirabilis* (Figure 2), whereas the laboratory reported only *P. mirabilis*. We retained this sample in the clinical sample comparison; we assessed agreement at the shared *Proteus mirabilis* level and reported the additional *P. aeruginosa* finding descriptively as a clinically relevant discordance.

Primary bacterial identification agreed well with laboratory results, with an accuracy of almost 90% (Table 4). Agreement in the plate isolate comparison group was ~10 percentage points higher than in the clinical sample comparison group given the greater frequency of polymicrobial growth in clinical samples. When staphylococcal taxa were grouped, agreement between in-clinic and laboratory results was almost perfect (98.3%), as six of seven disagreements were due to species-level discrepancies within *S. pseudintermedius*, *S. coagulans*, *S. felis*, and *S. schleiferi*. Only non-staphylococcal misidentification happened between *Enterobacter cloacae* complex and laboratory-reported *Klebsiella pneumoniae*.

Secondary bacteria were found in 15 clinical samples, and the agreement between in-clinic and laboratory results was low. The main reason is that the primary clinically relevant bacterial population often obscured lower abundance secondary bacteria, such as *Streptococcus* spp. and *Corynebacterium* spp. This resulted in secondary bacteria not being detected (*n* = 5, 33% of cases), or identifiable (*n* = 4, 27%) on direct culture.

### 2.2. Antimicrobial Susceptibility Testing (AST)

**Figure 3:**
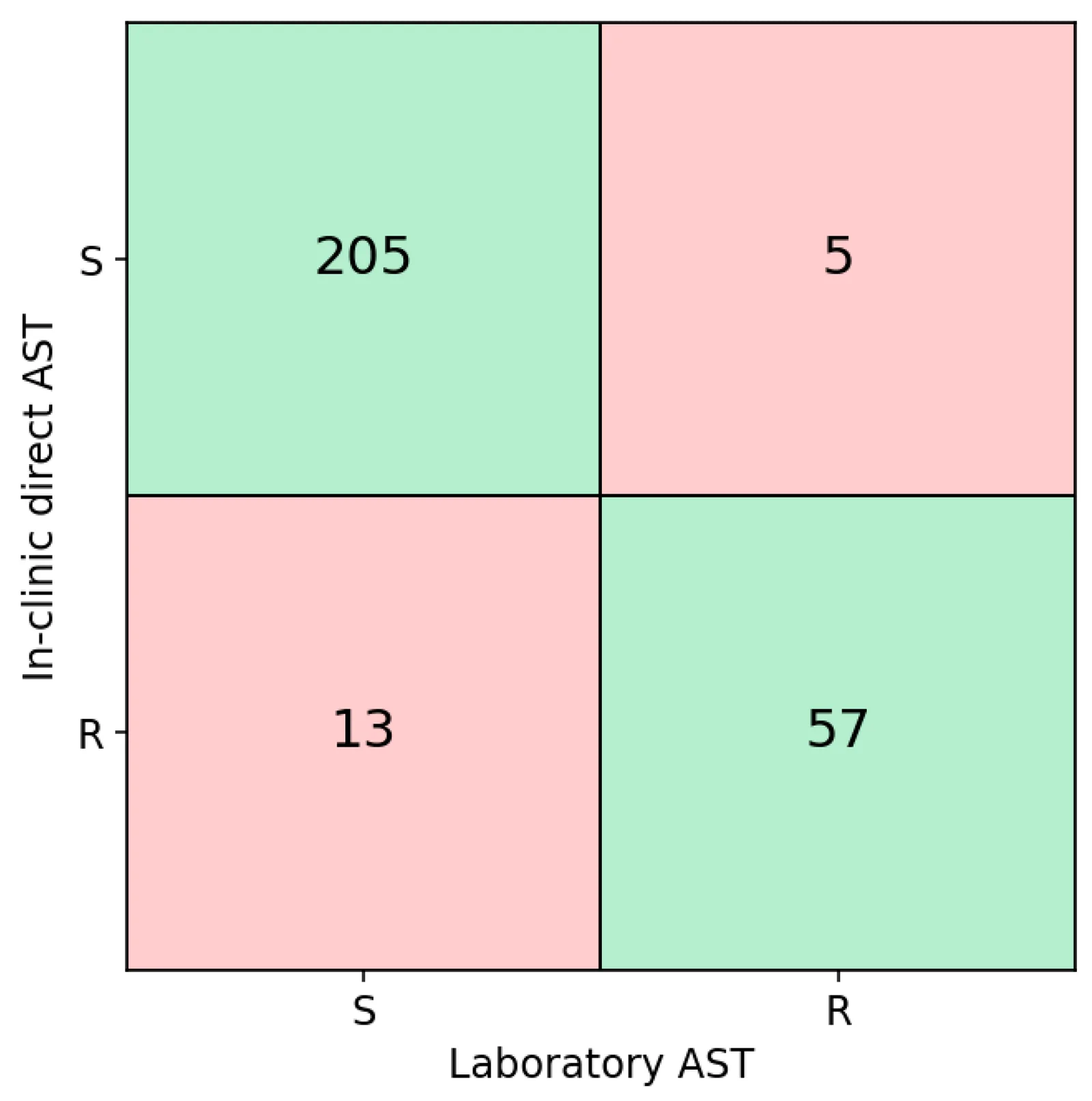
Confusion matrix for categorical agreement of antimicrobial susceptibility testing (AST) across all included antibiotic comparisons. Rows show in-clinic direct AST results and columns show laboratory AST results. Resistance (R) was treated as the positive condition, and susceptibility (S) was treated as negative.

Overall susceptibility agreement was 93.6% (262/280; 95% CI, 90.1–95.9%). Using the laboratory result as comparator and resistance as the positive condition, the direct AST workflow showed 91.9% sensitivity for detecting resistance (57/62; 95% CI, 82.5–96.5%) and 94.0% specificity for detecting susceptibility (205/218; 95% CI, 90.1–96.5%) (Figure 3).

Agreement was better in the clinical sample comparison group (126/131; 96.2%; 95% CI, 91.4–98.4%) than in the plate isolate comparison group (136/149; 91.3%; 95% CI, 85.6–94.8%), but there was no significant difference between groups (*p* = 0.14). AST categorical agreement was also similar across organ systems (*p* = 0.61), while separate agreements were 95.2% (119/125; 95% CI, 89.9–97.8%) for skin, 93.0% (40/43; 95% CI, 81.4–97.6%) for ears, and 92.0% (103/112; 95% CI, 85.4–95.7%) for urine.

Susceptibility disagreements were not concentrated in a single antibiotic but were distributed across several commonly tested antibiotic groups included in the study (Table 5). Most AST disagreements (12/18, 66.7%) were close to the interpretive breakpoint (<5 mm), whereas six disagreements (33.3%) were classified as clear disagreements. Of the six clear disagreements, only two had identifiable technical or reporting explanations. In one case, weak bacterial growth on the chromogenic plate may have affected zone interpretation. In another case, the laboratory reported polymyxin B susceptibility for a staphylococcal isolate, suggesting a probable reporting or transcription error.

Oxacillin results were available for 28 staphylococcal comparisons, including three laboratory-classified methicillin-resistant *S. pseudintermedius* (MRSP) isolates. VetBac classified all three MRSP isolates in this dataset as oxacillin resistant. However, the MRSP number was too small to draw conclusions about reliable MRSP detection, especially as we observed relatively large inhibition zones for β-lactam antibiotics in MRSPs. This may reflect the known unreliability of individual β-lactams for MRSP detection, but effects of the direct inoculation workflow, including inoculum density, medium, or other factors cannot be excluded.

## 3. Discussion

### 3.1. Culture

Identification workflow was strongest for dominant bacterial populations with distinctive chromogenic appearance or growth patterns (e.g., *E. coli*, *P. mirabilis*). Species-level differentiation was less reliable for visually similar staphylococci, and closely related *Enterobacter-Klebsiella* may also be difficult to separate visually on chromogenic medium. Therefore, direct identification should be interpreted as presumptive, with confirmatory laboratory identification recommended for ambiguous results and cases where species-level identification affects antimicrobial interpretation.

Regardless, bacterial identification showed very good agreement with laboratory results, particularly when visually similar staphylococci were grouped (98.3%, Table 4). The results are in line with the study on Flexicult Vet, in which the expert achieved 100% correct identification of bacterial species [13], but their dataset did not include visually similar species from staphylococci and enterobacteria. Compared with PoC tests (Uricult Vet, Speed Biogram) that report bacterial identification at the genus level, our performance was in the upper range of published results [15,16]. Thus, the high identification agreement observed in the present study suggests that the hybrid model preserved much of the expert-interpretation advantage while keeping test implementation in the clinic.

As indicated, most identification discrepancies were species-level differences within staphylococci. *S. pseudintermedius* and *S. coagulans* produced similar colony morphology and chromogenic appearance, while *S. felis* sometimes showed a less intense chromogenic reaction but not consistently enough for reliable separation. This limitation is relevant for precise taxonomy, epidemiology, and, in selected cases, for β-lactam AST interpretation, but less likely for non-β-lactam antibiotics, for which CLSI breakpoints are provided at the staphylococci level [18]. The only non-staphylococcal discrepancy was between *Enterobacter cloacae* complex and *Klebsiella pneumoniae*, which is plausible in a chromogenic medium-based workflow as these organisms produce similar colony morphology and chromogenic reactions and are difficult to separate visually [19].

Secondary bacterial population agreement was significantly lower than primary bacterial identification agreement, as expected for the direct culture workflow. Disagreements were mainly attributed to masking of lower abundance bacteria by the dominant population, under-recognition of secondary streptococci, and weak or non-specific chromogenic reactions. The clinical relevance of missed secondary bacterial populations varies by organ system. In urinary tract infections, secondary bacteria are not expected to be common, as infections are usually monomicrobial [20], although polymicrobial infections remain important in complicated cases [21]. The same applies to pyoderma where treatment is generally directed toward the predominant pathogen, supported by cytology, rather than toward every organism recovered in culture [5]. In otitis, secondary bacteria may be more relevant because mixed bacterial and yeast populations are common. Thus, culture results should be interpreted together with cytology, inflammation, underlying disease, and response to treatment.

Two additional discordant clinical sample cases illustrate an important limitation of comparing direct in-clinic culture with routine laboratory testing. In one urine sample, VetBac plate showed staphylococcal growth, whereas the diagnostic laboratory reported no bacterial growth. This discrepancy may reflect differences in sample handling and transport time, as urine culture results can be affected by pre-analytical handling conditions [22]. However, in the present study, all samples submitted to external laboratories were handled under routine conditions by the individual clinics. Therefore, we did not interpret this case as identification error. Another discrepancy occurred in an ear sample, where we detected *P. aeruginosa* alongside *Proteus* growth, whereas the laboratory reported only *P. mirabilis*. This difference is clinically important because *P. aeruginosa* is a major pathogen in canine otitis and may have a very challenging antimicrobial susceptibility profile [23].

### 3.2. Antimicrobial Susceptibility Testing (AST)

AST categorical agreement was high, generally above 90%, with comparable agreement between the clinical and plate isolate samples and across organ systems and antibiotic groups. Our AST results were broadly consistent with published evaluations of a few PoC tests that reported agreements above 90% [12,13] and were slightly higher than values of around 80–90% reported for some other approaches [14,16,17]. However, direct comparison with other PoC systems is limited because they use different susceptibility-testing workflows, mostly based on dilution-based methods. A recent bovine mastitis study used chromogenic Mueller–Hinton plates with disc diffusion and reported lower agreement than observed here, with overall agreement ranging from 73% to 89% [24]. However, that study used direct colony inoculation rather than direct inoculation from clinical samples, which may have contributed to smaller inhibition zones and lower agreement.

In our study, two-thirds of AST disagreements occurred near the interpretive breakpoint, suggesting that at least part of the discordance may reflect small differences in inhibition zone diameter rather than in antimicrobial activity. This is relevant because the VetBac workflow uses direct inoculation from clinical material rather than a standardized, pure 0.5 McFarland suspension [18]. Consequently, variation in inoculum density, interactions within mixed growth, and also digital zone margin readability could affect categorical interpretation near the breakpoint.

The clinical impact of disagreements also depends on direction: VetBac-resistant/laboratory-susceptible results can be treated as more conservative, whereas VetBac-susceptible/laboratory-resistant results could support use of an antibiotic classified as resistant by the laboratory. This situation occurred in five cases, representing 1.8% of all AST comparisons and 8.1% of laboratory-resistant results. Four of these five disagreements involved antimicrobials commonly considered for empirical or first-line treatment decisions, including ampicillin, amoxicillin–clavulanate, and trimethoprim–sulfamethoxazole. Although the absolute number of false-susceptible results was small, their potential clinical impact is important because they could affect antimicrobial selection.

Although all three MRSP isolates were correctly classified as oxacillin resistant, relatively large inhibition zones were observed around β-lactam discs. This finding may partly reflect the known limitation that individual β-lactam agents can produce false-susceptible results in mecA-positive *S. pseudintermedius*, making oxacillin the more reliable surrogate for MRSP detection [25]. However, inhibition zones were also observed around oxacillin discs, and colonies around the disc often showed weaker chromogenic coloration, suggesting that direct inoculation, medium/disc characteristics, or specific strains may affect AST readouts. A previous Flexicult Vet study [14] using chromogenic Mueller–Hinton agar reported similar concerns, with only 33.3% of MRSP isolates correctly detected. For more certain conclusions, a larger set of antimicrobial-resistant staphylococcal isolates should be evaluated systematically.

Despite these limitations, our results suggest that direct AST from interpretable chromogenic MH plates can provide clinically useful information on bacterial identification and susceptibility. The studied workflow may thus help bridge the gap between cytology/empirical prescribing and laboratory testing. Compared with fully clinic-interpreted chromogenic PoC systems, VetBac’s main practical distinction is its hybrid design: clinics perform sample inoculation, disk placement, incubation, image capture, and upload, while central staff perform bacterial identification and AST interpretation. This may reduce the need for microbiological expertise at the clinic level and limit operator-dependent interpretation, while preserving earlier access to culture-based information. However, complicated, non-responsive, or polymicrobial cases will probably still require standard laboratory confirmation, and local requirements for handling bacterial cultures in veterinary clinics may differ by jurisdiction.

### 3.3. Limitations

The study included only samples with interpretable bacterial growth on VetBac plates; therefore, these results should not be interpreted as diagnostic sensitivity or specificity for detecting bacterial infection across all submitted clinical samples from specific organ system infections. We did not include samples that were negative, showed weak growth, were contaminated, or were non-interpretable, as these are not suitable for direct culture and AST. Since the present study addressed conditional agreement after an interpretable direct culture result was available, the future studies shall have a prospective consecutive sample design and be performed separately for urine, skin, and ear samples. Infections in various organ systems differ substantially in expected bacterial load, polymicrobial growth, contamination risk, prior topical treatment, and interpretability.

Also, clinical suspicion of infection was determined by the attending veterinarian, and the study did not independently distinguish infection from colonization or contamination; therefore, bacterial detection should be interpreted as a microbiological finding within the submitted clinical context rather than as independent confirmation of infection.

Moreover, our methodology created an asymmetric comparison: we identified a case where the laboratory did not detect or report an organism visible on the VetBac plate, but it cannot quantify the opposite direction of disagreement. Another limitation is the modest sample size, particularly after grouping samples by organ system and antibiotic group.

Another limitation is that VetBac interpretation was performed by a single trained reader who was also the developer of the workflow. Although laboratory results were not available during VetBac interpretation, future validation should include independent blinded readers and inter-reader agreement assessment.

Finally, CLSI’s disk diffusion breakpoints [18] are based on standardized testing on conventional Mueller–Hinton (MH) agar, whereas we applied chromogenic Mueller–Hinton plates. Chromogenic Mueller–Hinton agar allows presumptive bacterial differentiation with AST on the same plate, which is practical for direct culture/AST workflows. However, its zone-diameter require further validation against conventional Mueller–Hinton agar as, to our knowledge, the suitability of chromogenic MH medium for AST has not yet been systematically evaluated. Therefore, potential differences between chromogenic and standard Mueller–Hinton agar may also contribute to discrepancies with conventional AST.

## 4. Materials and Methods

We collected clinical samples from dogs and cats with suspected bacterial skin, ear, or urinary tract infections during routine veterinary consultations at three private and one university veterinary clinic. Participating clinicians collected the samples and performed plate inoculation and incubation using a direct culture and AST workflow in their clinics.

The VetBac workflow consisted of five steps. (1) First, the clinical sample was inoculated directly onto chromogenic Mueller–Hinton agar in the clinic. (2) Antibiotic disks were applied according to the organ system panel, and plates were incubated overnight. (3) Plates were photographed on the VetBac calibration card (VetBac SIA, Riga, Latvia) and uploaded to the online platform. (4) Bacterial identification was performed centrally from the uploaded image based on colony color, morphology, growth pattern, and infection site. (5) AST was interpreted separately by measuring inhibition zones around antibiotic disks and assigning susceptible/resistant categories using CLSI VET01S Ed7 breakpoints [18] as the categorical reference framework when applicable.

Urine samples and moistened skin and ear swabs (Transport Swabs with Stuart liquid, VWR, Radnor, PA, USA) were inoculated directly onto ready-to-use chromogenic Mueller–Hinton (MH) agar plates (CHROMagar, Saint-Denis, France). We selected six antibiotic discs (BioMaxima, Lublin, Poland) per sample based on the organ system and applied them directly to the inoculated plates. The antibiotic panels mainly included drugs commonly used empirically for the respective infection types, with oxacillin included in skin/ear panels to support detection of methicillin-resistant *S. pseudintermedius* (MRSP).

Plates were incubated for 16–20 h at 35 °C in standard microbiological incubators. Samples without the expected chromogenic reaction or sufficient colony growth to support bacterial identification were excluded from further analysis. After incubation, the plates were imaged using the VetBac platform (VetBac SIA, Riga, Latvia). Each plate was placed on a calibration card, photographed with a smartphone, and uploaded to the online VetBac system. In parallel, each clinic submitted samples to the diagnostic laboratory normally used in their routine clinical practice.

Plate images were interpreted by the first author (B.C.), the developer of the VetBac workflow. The evaluator had access to sample type and infection site, which were required for interpretation, but laboratory identification and AST results were not available at the time of VetBac report generation. Bacterial identification was based on colony chromogenic reaction, according to the plate manufacturer’s instructions. For each sample, we recorded the primary clinically relevant bacterial population and, when noticeable, secondary bacterial populations. We defined the primary clinically relevant bacterial population as the population most relevant for interpretation based on infection site, dominant colony growth, chromogenic appearance, and expected clinical relevance. This designation was used due to polymicrobial growth, where not all visible bacterial populations are equally relevant for AST interpretation. Secondary bacterial populations were recorded when additional colony types were visible but were not considered the main target for direct AST interpretation. Inhibition zones were interpreted using CLSI VET01S Ed7 breakpoints as the categorical reference framework [18]. However, AST followed a direct disk diffusion workflow rather than a standardized procedure: samples were inoculated directly from clinical material without preparation of a standardized 0.5 McFarland suspension. Moreover, because laboratories did not report interpretive categories uniformly, AST analysis was restricted to a binary susceptible (S)/resistant (R) comparison; intermediate (I) or susceptible dose-dependent (SDD) interpretations, when present, were grouped with susceptible, i.e., non-resistant results.

For comparison, samples were split between two laboratory comparator approaches. In the clinical sample comparison group, the original clinical sample (i.e., skin/ear swab or urine) was submitted directly to the diagnostic laboratory in parallel with in-clinic testing. This group reflected the real-world diagnostic workflow agreement, where in-clinic and the laboratory may not always select or report the same bacterial population, especially in polymicrobial skin and ear samples. Secondly, in the plate isolate comparison group, a dominant bacterial population grown on a chromogenic agar plate was collected and submitted to the diagnostic laboratory to evaluate isolate-level agreement. The second group included mainly monomicrobial samples.

Routine diagnostic laboratory results were used as the comparator. Bacterial identification by the diagnostic laboratories was performed using MALDI-TOF mass spectroscopy. On the other hand, the specific AST methodology used by each diagnostic laboratory was not available and was therefore not standardized across samples.

All analyses were performed in the Spyder IDE 6.0.7 environment using Python 3.12.2. We calculated categorical agreement as the proportion of matching identified species and susceptible/resistant results among valid AST comparisons. Secondary bacterial population agreement was evaluated only for clinical sample comparisons, where complete agreement was scored as 1, partial overlap as 0.5, and no overlap as 0. Empty secondary fields on both sides were not counted as secondary bacterial population comparisons. Sensitivity and specificity assumed the laboratory result as the comparator and resistance as the positive condition. Proportions are reported with Wilson 95% confidence intervals (CIs). Differences between comparator groups were assessed using Fisher’s exact test.

## 5. Conclusions

In conclusion, the VetBac-based direct culture and AST workflow showed high agreement with standard laboratory results in interpretable canine and feline skin, ear, and urine samples. These findings support further evaluation of this hybrid in-clinic/centralized model as an adjunctive source of earlier antimicrobial decision support, pending confirmation by standard laboratory methods. Future prospective studies should determine the proportion of clinical samples from each organ system suitable for direct culture and AST and evaluate the workflow’s effect on antimicrobial prescribing decisions and clinical outcomes. Additionally, quality control validation of chromogenic Mueller–Hinton agar would also be valuable.

## Figures and Tables

**Table 1:** Performance of culture-based (phenotypic) point-of-care and in-clinic tests for bacteriuria detection, bacterial identification, and antimicrobial susceptibility testing (AST) in small-animal practice. For bacteriuria detection (BD), bacterial identification (ID), and AST, agreement (Agr) or sensitivity (Sens)/specificity (Spec) compared to laboratory methods are reported.

Test	Description; Samples Used	Culture	AST
Brookshire et al. [12]	Ampicillin-containing MH broth turbidity test; Canine urine	/	89.5%/100% ^Sens/Spec^
Flexicult Vet * [13,14]	Chromogenic MH plate with antimicrobial-containing compartments; Canine and feline urine	BD: 83%/100% ^Sens/Spec^ ID: 53–100% ^Agr^	89%/96% ^Sens/Spec^ 79.2% ^Agr^
Uricult Vet ** [15]	Dipslide culture system with CLED and MacConkey media; Canine urine	BD: 94–100% ^Agr^ ID: 42–84% ^Agr^	/
Speed Biogram *** [16]	Chromogenic multi-well assay with antimicrobials; Canine skin/ear swabs	ID ****: 100% ^Agr^, 0.53 (k-value)	81.7%/93.1% ^Sens/Spec^
PA-100 AST ***** [17]	Automated nanofluidic system; Canine and feline urine	BD: 87.1%/81.7% ^Sens/Spec^	88.7% ^Agr^

* SSI Diagnostica, Hillerød, Denmark, ** Aidian, Espoo, Finland, *** Virbac, Carros, France, **** primary-genus agreement only, ***** Sysmex, Kobe, Japan.

**Table 2:** Dataset flow for the agreement analysis.

Step	No. of Samples
Interpretable plates available for agreement analysis	60
Clinical sample comparison group (original sample sent to laboratory)	32
Plate isolate comparison group (dominant bacterium sent to laboratory)	28
Urine sample excluded (laboratory reported no growth)	1
Ear sample retained at *Proteus* level	1
Valid primary ID comparisons	59
Valid antibiotic-level S/R comparisons	280

**Table 3:** Distribution of bacterial taxa, as identified by external laboratories. Values represent the number of times each taxon was reported across all the included samples.

Taxa	Frequency
*Staphylococcus pseudintermedius*	27
*Escherichia coli*	11
*Streptococcus canis*	9
*Proteus mirabilis*	7
*Enterococcus faecalis*	6
*Corynebacterium* spp.	5
*Staphylococcus coagulans*	4
*Staphylococcus felis*	3
*Staphylococcus schleiferi*	2
*Staphylococcus* spp.	2
*Pseudomonas aeruginosa*	1
*Streptococcus* spp.	1
*Pasteurella multocida*	1
*Enterobacter cloacae* complex	1
*Klebsiella pneumoniae*	1

**Table 4:** Bacterial identification agreement (in %) between in-clinic and laboratory results by comparator group (clinical sample, plate isolate) and organ system (skin, ears, urine). Species-level agreement was calculated using the exact reported bacterial taxa. Staphylococci-pooled agreement treated staphylococcal taxa as one group. Secondary bacterial population agreement was evaluated in clinical samples when additional bacterial populations were reported alongside the primary clinically relevant populations.

	All	Clinical	Plate Isolate	Skin	Ears	Urine
**Primary bacteria**						
Species-level	88.1	83.9	92.9	84.0	81.8	95.7
Staphylococci-pooled	98.3	100.0	96.4	100.0	100.0	95.7
**Secondary bacteria**						
Species-level	16.7	16.7	/	22.2	10.0	0.0

**Table 5:** Distribution of antimicrobial susceptibility testing (AST) categorical agreements in % by antibiotic group.

Antibiotic Category	AST Agreement (%)
Beta-lactams (*n* = 129)	95.3
Fluoroquinolones (*n* = 71)	91.5
Clindamycin (*n* = 25)	92.0
Trimethoprim–sulfamethoxazole (*n* = 21)	95.2
Others (*n* = 34) *	91.2

* Erythromycin, fusidic acid, neomycin, polymyxin B, florfenicol, gentamicin, doxycycline.

## Data Availability

The data presented in this study are available on request from the corresponding author due to commercially sensitive and proprietary information related to the VetBac workflow and source data from routine diagnostic laboratory reports. Requests will be reviewed case by case and may require approval by the data holder and/or a data sharing agreement.

## Abbreviations

The following abbreviations are used in this manuscript:

AST	Antimicrobial Susceptibility Testing
CI	Confidence Interval
CLSI	Clinical and Laboratory Standards Institute
CLED	Cystine–Lactose–Electrolyte Deficient
ID	Identification
ISCAID	International Society for Companion Animal Infectious Diseases
LAMP	Loop-Mediated Isothermal Amplification
MH	Mueller–Hinton
MRSP	Methicillin-Resistant *Staphylococcus pseudintermedius*
PoC	Point-of-Care
QC	Quality Control
S	Susceptible
R	Resistant
SDD	Susceptible Dose Dependent
UTI	Urinary Tract Infection
